# Development of a conceptual model of BKV impacts on health-related quality of life in kidney transplant recipients: a qualitative study

**DOI:** 10.1186/s41687-025-00987-x

**Published:** 2026-01-07

**Authors:** Courtney N. Hurt, Maja Kuharic, Sara Shaunfield, Juergen Beck, Alex Bastian, Kevin Fowler, Emilie Jaeger, Marcus May, Erik van den Berg, John Friedewald, John D. Peipert

**Affiliations:** 1 Medical Social Sciences, Northwestern University Feinberg School of Medicine, Chicago, IL USA; 2 Memo Therapeutics, Schlieren, Switzerland; 3 The Voice of the Patient, Inc., Saint Louis, MO USA; 4 Comprehensive Transplant Center, Northwestern University Transplant Outcomes Research Collaborative, Feinberg School of Medicine, Chicago, IL USA; 5 Centre for Patient Reported Outcomes Research, University of Birmingham, Birmingham, UK

**Keywords:** Qualitative research, BKV, Kidney transplant, Conceptual model, Patient-reported outcomes, Health-related quality of life

## Abstract

**Background:**

BK virus (BKV) is a common latent virus that can reactivate in kidney transplant recipients due to immunosuppressive therapy, potentially leading to graft dysfunction or loss. While clinical management of BKV is well studied, little is known about its broader impact on patients’ daily lives and well-being. No conceptual model currently exists to describe the health-related quality of life (HRQoL) impacts of BKV from the patient perspective.

**Methods:**

We conducted a qualitative study using semi-structured concept elicitation interviews with 12 adult kidney transplant recipients who had experienced BKV reactivation. Participants were recruited using purposive sampling to ensure diversity in demographics and clinical experiences. Interviews were transcribed and analyzed using thematic analysis with iterative coding, saturation tracking, and structured impact prioritization to develop a conceptual model of BKV-related HRQoL impacts.

**Results:**

Participants described a range of psychosocial and practical challenges associated with BKV, despite the virus often being asymptomatic. Key themes included emotional distress, fear of graft loss, confusion about treatment, disruption to work and daily routines, and increased burden of care coordination. Many participants reported feeling unprepared and unsupported, often needing to advocate for themselves within the healthcare system. These experiences were synthesized into a conceptual model illustrating the multidimensional impact of BKV on HRQoL.

**Conclusions:**

This study presents the first patient-informed conceptual model of BKV-related HRQoL impacts in kidney transplant recipients. Findings highlight the need for improved patient education, communication, and support strategies. The model provides a foundation for future development of patient-reported outcome measures and interventions that address the unique burdens of BKV in transplant care.

**Supplementary Information:**

The online version contains supplementary material available at 10.1186/s41687-025-00987-x.

## Abstract (plain language summary)

Most people are exposed to a virus called BKV virus (BKV) during childhood. It usually stays inactive in the body and does not cause problems. But for people who receive a kidney transplant, the medications they take to prevent rejection can weaken their immune system. This can cause BKV to “wake up” and become active again. When this happens, it can damage the new kidney and may even lead to its failure. There is no approved treatment for BKV, and managing it often involves reducing the very medications that protect the transplant. Although BKV often doesn’t cause physical symptoms, it can still have a major impact on people’s lives. In this study, we spoke with 12 kidney transplant recipients who had experienced BKV. They described how the virus affected their mental health, work, relationships and ability to manage their care. Many felt scared about losing their kidney, confused about treatment, and overwhelmed by the need to coordinate appointments and insurance on their own. In most cases, the burden came not from feeling sick, but from the emotional strain and extra care required to manage the condition. This study is the first to create a model that shows how BKV affects quality of life. It helps highlight the emotional and practical challenges patients face, not just the medical ones. We hope this work leads to better support, education, and care for people living with BKV, and helps researchers develop tools that reflect what matters most to patients.

**Supplementary Information:**

The online version contains supplementary material available at 10.1186/s41687-025-00987-x.

## Introduction

BK virus (BKV) is a human polyomavirus that poses a risk to kidney transplant recipients, potentially leading to allograft dysfunction. The majority of the general population acquires BKV asymptomatically during childhood and the virus remains latent in renal tissue and the urinary tract [1]. In immunocompromised individuals, particularly kidney transplant recipients undergoing immunosuppressive therapy to prevent graft rejection, BKV can reactivate and become an opportunistic pathogen [2]. Such reactivation can lead to BK viremia, occurring in 10–30% and BKV-associated nephropathy (BKVN), in 1% to 10% [3–5]. Before widespread adoption of BKV screening post-transplant, initial outcomes following a BKVN diagnosis were poor, with graft loss rates reaching up to 50% within five years [6]. Although screening and monitoring have improved outcomes, BKV remains a complex and burdensome condition to manage.

Clinical management of BKV introduces distinct logistical and emotional concerns that differentiate it from general post-transplant challenges. Currently, there is no FDA-approved antiviral treatment for BKV infection. The primary management strategy involves reducing immunosuppression to enhance the patient’s antiviral immune response and decrease viral replication [6]. Because BKV treatment relies on reduction of immunosuppression, patients and providers need to navigate a delicate balance between viral control and graft preservation. This therapeutic ambiguity may serve to heighten anxiety and uncertainty among patients [7]. Additionally, the need for increased monitoring may also contribute to additional psychosocial stressors post-transplant, including financial burdens [8]. However, patient perspectives on the broader effects of BKV, including psychological, social, and practical impacts, remain poorly understood [9]. Although qualitative examinations of patient experiences of graft failure [10] and post-transplant self management [11] may be found, existing literature lacks comprehensive studies directly examining the burden of illness experienced by post-transplant patients with BKV, including effects on mental and physical well-being, daily functioning, and overall health-related quality of life (HRQoL).

A conceptual model is a visual representation outlining how specific biological and psychosocial factors related to a condition are linked to outcomes, allowing for prioritization of essential elements for study and intervention [12]. Concept elicitation interviews, which gather in-depth qualitative data directly from patients, are useful to summarize patients’ perceptions and the multidimensional impact of their disease to inform conceptual model development. Interviews involve discussions with patients, exploring their experiences, perceptions, and various dimensions of disease burden, capturing nuanced and subjective aspects of HRQoL that may be overlooked by quantitative or lab-based measures. While several models have described HRQoL of transplant recipients broadly, none exist for BKV. Given BKV’s emotional and practical impacts, a disease-specific conceptual model is needed to guide future research, patient education, and interventions.

We conducted a qualitative research study using semi-structured concept elicitation interviews with 12 kidney transplant recipients with a history of BKV in order to develop the first conceptual model of BKV-specific HRQoL impacts. Well-established qualitative methods including purposive sampling, iterative coding, saturation tracking, and structured approaches to impact prioritization were used to gather, analyze, and synthesize data from a small sample. The objective of this study was to generate an initial conceptual model to inform and improve care for kidney transplant recipients affected by BKV. Given the exploratory nature of this study, findings represent an initial step in understanding the disease burden of BKV, providing a foundation for future research, including the development of patient-reported outcome measures, and targeted interventions to address specific needs of kidney transplant recipients affected by BKV.

## Materials and methods

### Study design

The study was approved by the Northwestern University Institutional Review Board (STU00221982). Participants provided informed consent. Reporting follows Consolidated Criteria for Reporting Qualitative Research (COREQ) guidelines (see Supplemental Files) [13]. 

We drafted a semi-structured interview guide based on prior qualitative research in conceptual model development, content validity, treatment experience, and symptom assessment [14–17]. Early patient input and interview feedback informed refinements (Supplemental Files). Interviewers used open-ended questions and probes to explore BKV’s impact on participants’ lives, functioning, emotional well-being, and healthcare experiences. Participants were also asked to identify an analogy for their BKV experience to facilitate expression and encourage thoughtful reflection. At the end of the interview, participants generated a list of discussed impacts and rated each on a 0–10 scale of health-related quality of life (HRQoL) impact, where 0 represents “No impact at all” and 10 represents “Extreme impact.”

### Participant selection

Eligibility criteria included: (1) age ≥ 18; (2) self-reported BK viremia or DNAemia post-kidney transplant; (3) English fluency; and (4) capacity to consent. Individuals with significant cognitive impairment that would preclude meaningful participation in a qualitative interview, as judged by study staff were not eligible. Participants were referred by a patient advocate affiliated with Memo Therapeutics, and a transplant nephrologist based at Northwestern. Purposive sampling was used to create a diverse sample, prioritizing regional and racial representation to reflect lived experiences of patients in transplant centers with potentially varying BKV treatment protocols. A study team member contacted potential participants, conducted a verbal eligibility screener, and scheduled virtual interviews following e-consent in REDCap. Participants received a USD $75 electronic Visa gift card following the interview.

### Data collection

The interview guide was developed using patient input and designed to explore patients’ experiences across several domains, including symptom, diagnosis, and treatment experiences, emotional and psychosocial impacts, daily life disruptions, disease education, and interactions with clinicians. Our approach to interview guide development and the guide itself can be found in the Supplemental Files. Twelve 60–90 min interviews were conducted from August to October 2024. Participants completed a REDCap-based demographic and clinical questionnaire [18, 19]. Master’s-level researchers (EJ, CH) with qualitative training conducted interviews via Zoom, which were audio-recorded, transcribed, and de-identified. Interview field notes were formatted in Excel for rapid analysis and codebook development. Saturation was evaluated after six interviews, continuing until 12 were completed to ensure representation [20].

### Data analysis

An audit trail documented detailing team discussion and coding decisions [21]. The preliminary codebook was developed based on rapid inductive analysis [22] of field notes including responses to open-ended questions and experience analogies from the first three interviews. Using an applied thematic analysis approach, two analysts (CH and EJ) used the preliminary codebook to independently code new interview transcripts using the comment feature in Microsoft Word, meeting between each coding to evaluate agreement, discuss discrepancies, and further refine the codebook. This was conducted a total of three times, at which point, the analysts achieved ≥ 70% agreement (Krippendorff’s alpha > 0.70) [23]. Discrepancies were resolved by team consensus and finalized codes were applied to all transcripts using Dedoose qualitative analysis software [24]. Constant comparison [25] and memoing were used to identify subthemes. Codes were only applied to impacts that the participant attributed to the experience of BKV infection. Impacts related to general post-transplant status, or other attributions, were not coded.

Salient BKV impacts were identified based on frequency and average HRQoL impact ratings. Themes and subthemes most commonly rated and identified as most detrimentally impactful to HRQoL were included in the final conceptual model, which builds upon Wilson and Cleary’s established HRQoL framework [26]. The model development process began with the integration of primary themes and subthemes, organizing them into broader domains representing distinct aspects of BKV burden, and prioritizing those most salient in the dataset. We established criteria to identify concepts for the final conceptual model:

1. The theme or subtheme was endorsed by ≥ 25% of participants (*n* ≥ 3) during the HRQoL ratings discussion; and
2. The mean HRQoL rating was ≥ 4 (on a scale of 0 to 10)

Given the exploratory nature of this qualitative study and the small sample size, we opted for an inclusive threshold for salient concepts. We defined a threshold of ≥ 25% (i.e., endorsement by at least 3 participants) to capture recurring and meaningful concepts. This approach is consistent with qualitative research practices prioritizing depth and nuance of patient experience over statistical generalizability so that impactful, but less common themes are not prematurely excluded from model development [27]. Descriptive statistics (frequency, mean, range) were used to characterize participant demographics and clinical data.

## Results

Concept elicitation interviews were conducted with 12 participants, who were diverse in age, gender, and race/ethnicity. Interviews were an average duration of 90 min, ranging from 45 min to 120 min. The sociodemographic and the health characteristics of the participants are summarized in Tables 1 and 2. Details on participants’ support systems can be found in Table S1 in the Supplemental Files.

**Fig. 1:**
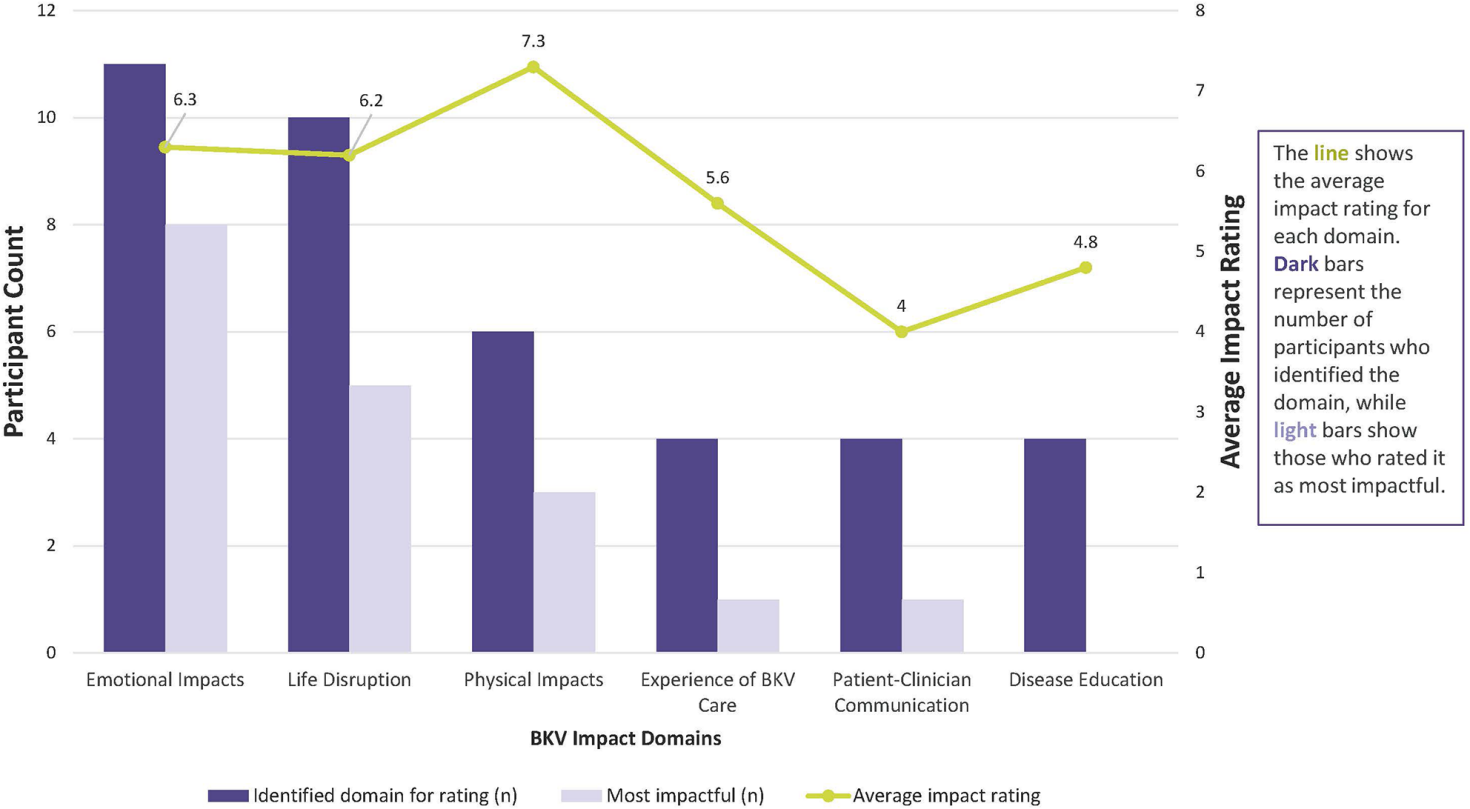
BKV Impact domains- average ratings and most impactful

Seven themes were identified: Social and Caregiver Support, Emotional Impacts, Experience of BKV Care, Physical Impacts, Patient-Clinician Communication, Disease Education, and Life Disruption. Because BKV can affect both transplant recipients and their support systems—sometimes directly and sometimes through the support systems mitigating its impact—the Social and Caregiver Support theme is interwoven with the other six themes, and its influence is reflected in their context. Thematic saturation was identified after the fifth interview. An additional seven interviews were completed to ensure comprehensive capture from a diverse sample. Overall frequencies, rating frequencies, example quotes, and HRQoL impact ratings of salient themes and subthemes can be found in Tables 3, 4, 5, 6, 7 and 8 and in Fig. 1. Additional results for subthemes not salient can be found in Tables S2-S7 in the Supplemental Files.

### Disease education

Participants generally reported they had no pre-transplant education about the possibility or the risks of BKV. In addition, some indicated that they received no educational materials at diagnosis. As a result, many felt compelled to educate themselves about BKV. This self-directed learning ranged from seeking information from friends and family to internet searches and more rigorous research efforts. For some, the process provided clarity and empowerment. Learnings were shared with clinicians. One participant described, “*I tried to study about the virus and the effects*,* and when I found out about it*,* I would [look at] my lab work*,* and compare the values from one week to the next. I made a decision not to allow [BKV] to make me fearful. I made a conscious decision that I may have it*,* but I trusted those that I’m in care with*.” For others, it created confusion, anxiety, and additional stress, further impacting their overall well-being. One participant noted, “*As I read everything about BK virus*,* it was scary for me. I was stressed out. I worried whether it’s gonna affect my transplant and whether it’s gonna persist [as we were] reducing the immunosuppressant.*”

### Emotional impacts

A new BKV diagnosis was an emotional challenge for many participants. One participant used an analogy of finding a monster under their bed when describing what it was like to be diagnosed with BKV, adding, “*there’s something threatening me in a place where I should feel safe.”* A lack of education prior to diagnosis could heighten the emotional impact, as another participant said being diagnosed was like finding out they have cancer because they had never heard of BKV prior to their diagnosis. Emotional Impacts related to complexities and uncertainties in managing BKV were also commonly reported. A predominant subtheme is a fear of graft loss. As one participant shared their experience being treated for BKV while in the hospital, “*The fear of the doctor coming in and telling me my kidney has rejected*,* and I have to go back on dialysis was something that I feared more than dying.*” Uncertainty about treatment may compound this emotional burden, as some participants reported conflicting opinions from clinicians and unclear pathways for viral management, leaving them unsure about the efficacy or long-term implications of their care. Fear of graft loss, stress, frustration and uncertainty about treatment may loom large, particularly when test results show elevated levels of BK virus, leading to a sense of vulnerability and helplessness. “*Banging my head against the wall*” is the analogy one participant chose to describe frustration with BKV monitoring and explained, “*It’s just over and over, the labs. Numbers up, numbers down. It’s just constantly… I just want to be like, ugh!*” Anxiety about lab tests is another common subtheme, as participants described a sense of dread around needles or receiving results that might indicate worsening conditions or necessitate changes in their treatment plan. One participant even sought out psychological support to manage their anxiety around ongoing BKV labs.

While BKV may exert an emotional toll on kidney transplant recipients, this may also extend to their family members and care partners who often share in the worry and witness their loved one facing fears of graft loss, uncertainty about treatment, and the stress of navigating a complex fee-for-service healthcare system. One participant described impact on her husband, “*We were just so worried. He’s the main care person. He gets me through all of this*,* and it was a strain on him to know*,* ‘Okay*,* we got one more thing we gotta figure out.’*”

Importantly, this emotional burden can be bidirectional. Participants reported feeling worse when they observe the worry and distress of their family members and caregivers, compounding their own feelings of frustration and helplessness. This dynamic may create a feedback loop where the emotional struggles of both the patient and their support network intensify each other. The logistical challenges of frequent travel, coordinating appointments, and managing insurance may further add to this shared burden. Living donors, in particular, may experience a unique emotional burden as they grapple with feelings of responsibility for the transplant’s success. Or, living donor recipients with BKV infection may feel additional pressures, like one participant noted regarding her living donation from a friend, “*I am so grateful to her*,* and our whole relationship is tied up with this*,* too. I think if I were to lose or damage the kidney*,* then there’s this element where I feel like*,* she gave me this incredible gift and that’s my responsibility to take good care of it. So*,* I would feel kind of guilty if anything were to happen.*”

Reducing immunosuppression also appears takes away a sense of control the patient has over their transplanted kidney and is at odds with the emphasis on medication adherence. The duration of treatment is often undefined, and participants described a growing impatience at the time required to see improvement or resolution. Other reported emotional impacts stem from a sense of futility or loss of control over treatment itself. One participant reported, “*Once I learned that it could affect the kidney transplant*,* my emotions were all over the place. I felt sad. I felt defeated. I asked*,* “Is it something that I can do more? Do I need to take more water? Do I need to eat something different?’ And they was like*,* ‘No. It’s pretty much out of your control.’ That really tore me down because this was my second transplant and I feel*,* ‘This one has to work. I may not be able to get transplanted again.’*”

### Life disruption

Participants commonly reported BKV-related disruptions in finances, travel, time, and work. Financial strain for these US-based participants was a concern, as out-of-pocket costs, including copays and deductibles, for treatment and monitoring could be substantial. One participant shared, “*After March of next year I’m off COBRA and I’ll be on Obamacare and it’s $1*,*000 a month for insurance and then who knows what I’ll have to pay on top of that because the deductible is so high. So*,* the cost of labs*,* they’re $2*,*000 to $2*,*500 a visit and go once a month*,* that’s a lot of money.”* Burdens relating to travel included both restrictions on leisure travel and inconvenient commutes for BKV care. Variability in reporting BK viral loads across lab sites further constrained travel for participants, requiring some to remain close to home to ensure reliable results. Managing BKV also imposed significant time demands. Participants described the burden of frequent appointments, tests, and ongoing communication with clinicians, which detracted from social responsibilities and emotional well-being. While most were able to integrate BKV monitoring and treatment into their daily schedules, minor disruptions such as scheduling conflicts with work or school were common. Although these adjustments were largely reported to be manageable, they still caused stress and inconvenience for some. While reflecting on the need to coordinate lab appointments and determine out of pocket costs for treatment, one participant said, “*It was frustration*,* and stress*,* and time having to figure all that out and fight about it.”*

### Experience of BKV care

Additional, or complex care coordination, often requiring a degree of independent navigation, including arranging for care or testing closer to home or seeking treatments not available through their current nephrologist were commonly reported. Participants also described inconsistencies in care, for example, receiving conflicting lab values for the same test across different facilities, complicated BKV management. Some were compelled to limit where they could have their BKV levels drawn to ensure their nephrologist’s confidence in the accuracy of the results. Some participants reported issues with laboratory orders, meaning that their BKV titers were not collected with their other labs, necessitating additional coordination to get required tests. Problems with navigating insurance coverage for BKV treatment and monitoring were also identified as burdensome. No participants reported a complete inability to obtain BKV treatment or monitoring due to issues with their health insurer, but there was reported variation in how easily or quickly they were able obtain these approvals. Some participants described delays in care and monitoring while waiting for insurance approvals, to the extent that when one participant presented for their IVIG treatment, they were given the choice to pay out of pocket (USD $15,000+) or return once their insurer had given final approval. Another participant described their experience with delayed care, “*I was frustrated and concerned because the way it was brought to me was*,* ‘We need to get treatment started immediately. ‘Cause your kidney is in jeopardy.’ So*,* I was anxious ‘cause I’m like*,* ‘Okay*,* what’s the holdup?’ I was calling to check to see if things have gone through. I was concerned and frustrated by it.*”

Many participants expressed gratitude for having health insurance, recognizing it as a decisive factor in managing and treating their BKV. They acknowledged that without this coverage, the challenges they faced, such as navigating multiple providers, undergoing frequent lab tests, and accessing specialized treatments, would be more burdensome. Participants were acutely aware of the potential strain that individuals with lower coverage might endure. Participants often discussed their issues with health care system navigation and care coordination alongside discussions of Emotional Impacts and Life Disruptions, suggesting a multifaceted impact these challenges bring to patients’ lives.

### Physical impacts

Many participants reported that their treatment was ultimately effective at reducing or eliminating BKV, and a majority reported they had no negative clinical outcomes or symptoms related to their BK virus infection. However, when physical impacts were reported, they largely fell into two categories—those participants attributed to BKV itself and those participants attributed to BKV treatments.

Of those who experienced physical symptoms that they attributed to BKV, symptoms resembled non-specific viral infection, including fatigue, diarrhea, weight loss, and fevers. One participant had mild physical discomfort in the region of the transplanted kidney that correlated with increased viral load. Physical impacts that participants attributed to BKV treatment were more common, including muscle weakness, headaches, and hyperglycemia. Physical Impacts frequently co-occurred with Emotional Impacts, especially fear of graft loss in both the participant and their support system. Some participants reported limitations on socialization and travel caused by the physical impact which, in turn, affected their emotional well-being.

### Patient-clinician communication

Some participants described minimal or ineffective communication regarding BKV from their clinician. These participants reported that the communication caused misunderstandings or was not reassuring. Some perceived a contradiction between standard immunosuppression to maintain a transplanted kidney and reduction of immunosuppression to treat BKV. As one participant shared, “*When you get the transplant*,* they stress the importance of taking those immunosuppressants*,* just how important they are if you wanna keep your kidney. ‘You take them every single day*,* twice a day if we tell you. Take them at the same time.’ So*,* it was just a big thing to come off them*,* because I was like*,* ‘They stressed the importance of it. They said I have to take them if I wanna keep my kidneys. Now*,* they don’t care if I lose the kidney.*’” However, for others, more education and communication with their nephrologist served to foster confidence in the treatment plan and alleviate some of their fears.

### Conceptual model

**Fig. 2:**
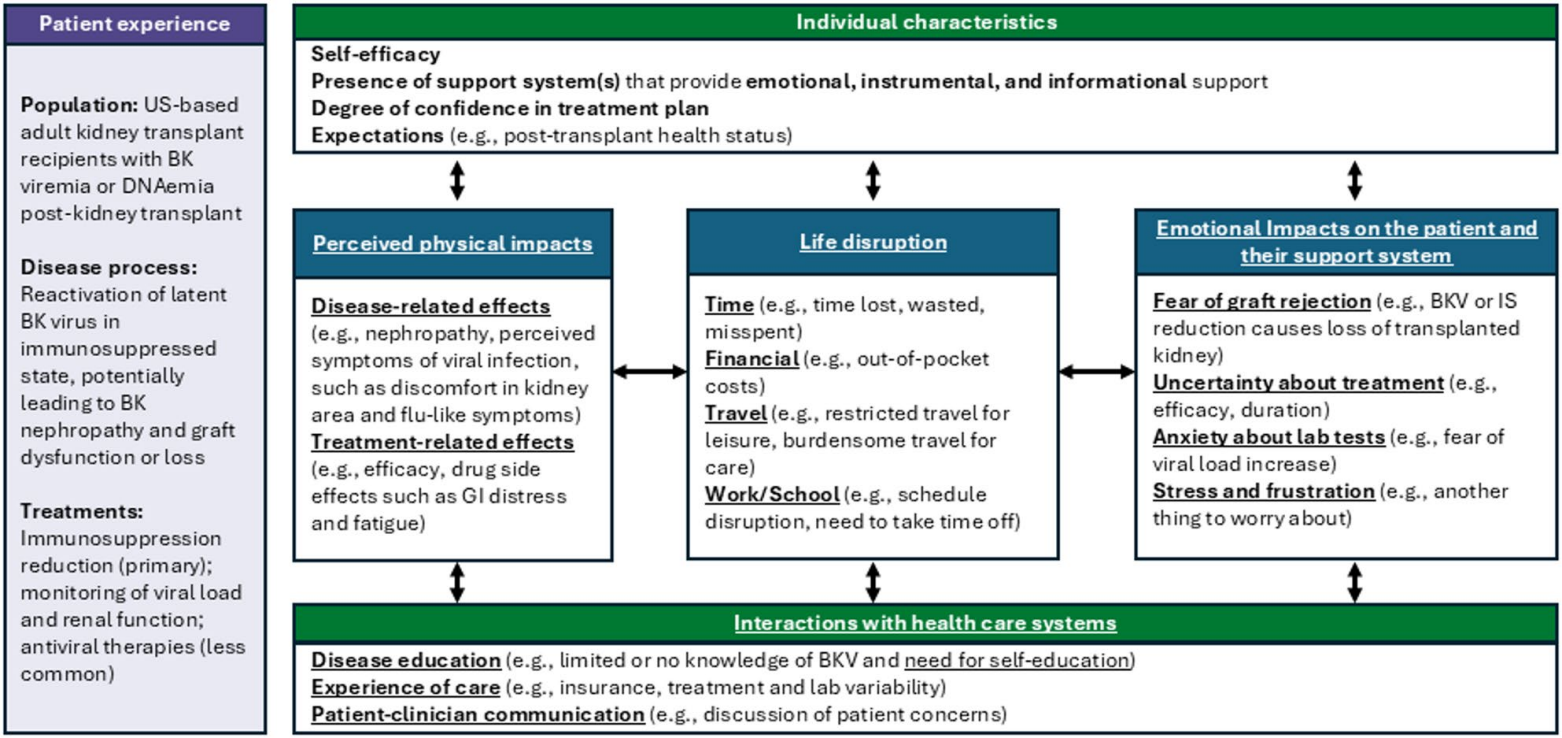
Conceptual model of impacts of BKV on HRQoL in kidney transplant recipients

A conceptual model was developed illustrating the multifaceted impact of BKV on kidney transplant recipients, based on the themes and subthemes rated highest in both frequency and impact (Fig. 2). The model visually represents the interconnections between salient impacts, categorized into four major areas:

- **Perceived physical impacts**, comprised of symptoms and health challenges participants attribute to the BK virus or treatment
- **Life disruption**, reflecting broad impacts on daily activities and work
- **Emotional impacts** on the patient and their support system
- **Interacting with health care systems**, including challenges related to navigating complex care pathways, learning about BKV, and clinician communication, with disease education and patient-clinician communication representing factors more within a clinician’s locus of control, rather than an experience of BKV care affected by external factors like interpretation standards across laboratories, insurance barriers, and variable treatment guidelines

Parenthetical examples for each salient theme or subtheme (indicated with underlined text in Fig. 2) add context but are not intended to be inclusive of all details reported by participants. The model also includes an *Individual characteristics* section indicating personal factors that might amplify or buffer the impacts identified in other domains. While the relationship beyond these individual characteristics and the broader impacts of BKV is beyond the scope of this study, their inclusion underscores a nuanced and individualized nature of disease experience as interpreted from these qualitative interviews.

## Discussion

This qualitative study presents the first conceptual model of the impact of BKV infection in kidney transplant recipients. Using a patient-centered approach, we identified interconnected impacts beyond clinical manifestations, including practical and psychosocial effects that can be shaped by healthcare interactions and individual characteristics. This conceptual model addresses a gap in transplant literature by mapping the complex, interconnected nature of BKV-related impacts, including moderators and mediators, in order to provide a framework to guide both clinical assessment and intervention development.

Existing frameworks of post-transplant experiences often focus on general challenges and stressors, including immunosuppressive medication adherence, and routine follow-up [28]. Our findings suggest that BKV introduces additional complexities. Emotional impacts, as fear of graft loss, uncertainty regarding viral activation, and treatment ambiguity were prominent. While such concerns align with prior reports of distress in transplant populations [29, 30], these emotions may be heightened by the unpredictable nature of BKV.

Participants also described difficulties in care coordination and insurance processes, contributing to emotional distress and life disruption. Although these factors were not formally evaluated, they appear to intensify patient burden. Clarifying care pathways and improving communication may help but require further study.

Although we did not assess clinical outcomes, some participants expressed concern about balancing BKV treatment with the need to remain immunosuppressed preserving graft function. These concerns highlight a potential area for patient education and shared decision-making, particularly around the risks and rationale for immunosuppression adjustments.

Participants frequently reported that they were unfamiliar with BKV before diagnosis and found clinical information difficult to access or understand. This underscores the potential value of improved educational materials and peer support programs, which have shown benefit in other contexts [31, 32]. Our findings support further research into the development of tools that can better prepare patients for managing post-transplant infections like BKV.

The impacts reported here do not definitively point to concepts that would be incorporated as endpoints in clinical trials. While many of the salient impacts relate to psychosocial wellness and practical life disruption, which are concepts not typically prioritized for primary endpoints in registration trials with the US FDA [33], this conceptual model demonstrates that patient-centered outcomes in BKV extend beyond viral clearance. Specifically, impacts related to immunosuppression modification, potential for medication non-adherence, and treatment side effects may be important to consider in trial design. For post-market or comparative effectiveness research, outcomes such as fear of transplant rejection, healthcare navigation burden, and quality of information provision may be particularly relevant. Understanding these patient priorities can inform both trial endpoint selection and design of supportive care interventions.

This study’s strengths include the use of rigorous qualitative methodology and the integration of literature review, research partner input, and patient interviews to create a data-driven conceptual model. This model may inform the future development of BKV-specific PRO measures or educational tools, though further research is needed to explore these applications. For PRO measure development, the model is a foundation providing candidate concepts (e.g., fear of graft loss, care coordination burden) that could be operationalized into BKV-specific items or scales. The interconnected nature of impacts suggests that comprehensive assessment would require multi-domain measurement rather than single-construct approaches. For clinical practice, the model highlights actionable targets for intervention, including the need for proactive patient education about BKV at the time of transplant (before diagnosis), structured communication protocols around immunosuppression changes, and care coordination support. For research, the model provides a hypothesis-generating framework for investigating relationships between healthcare system factors and patient outcomes. It also identifies priority areas for intervention studies including testing the impact of early education or peer support programs on reducing fear and uncertainty.

Several limitations should be considered when interpreting our findings. Most participants (67%) resided in the Midwest region of the U.S., potentially limiting generalizability to other regions, though there was diversity in terms of represented transplant centers. Participants were based in the U.S. and had health insurance that ultimately covered most or all BKV management costs, though financial stressors related to these actual or potential costs were salient. While financial impacts related to the cost of care are mostly relevant to patients in the U.S., it is possible that the other life disruption themes (travel, taking time off of work) can contribute to financial stressors in other countries even with universal health coverage. More research is needed to understand how life disruptions attributed to BKV infection and treatment may vary globally. The sample was also highly educated, with two-thirds of participants holding advanced degrees, which may not reflect the experiences of individuals with different educational or socioeconomic backgrounds who might face additional challenges in navigating healthcare systems or understanding medical information. Despite the racial and ethnic diversity of our sample (75% non-white participants), we did not observe notable differences in reported experiences across racial or ethnic groups. This may reflect the sample’s relatively high education level, though the small sample (*n* = 12) limits our ability to detect meaningful differences that may exist in broader populations. Additionally, some participants were recruited through patient advocacy groups, potentially reflecting a more activated patient population. Future studies should include broader populations and non-English speakers.

This study developed the first conceptual model of the burden of BKV in kidney transplant recipients, revealing interconnected impacts across physical, emotional, and life domains, mediated by healthcare system interactions and individual patient characteristics. These findings indicate that BKV introduces challenges beyond routine transplant care, including the need to navigate complex treatment protocols and cope with psychological uncertainty.

**Table 1:** Participant sociodemographic characteristics (*N* = 12)

Sociodemographic Characteristic	*n* (%)
Age, mean (range)	48 (31–66)
Age categories	
< 40 years	3 (25%)
40–49 years	3 (25%)
50–59 years	4 (33%)
≥ 60 years	2 (17%)
Gender, n (%)	
Women	7 (58%)
Men	5 (42%)
Race/Ethnicity, n (%)	
Black or African American	4 (33%)
Asian	3 (25%)
White	3 (25%)
American Indian or Alaska Native	1 (8%)
Hispanic or Latino	1 (8%)
Education Level, n (%)	
Advanced degree (MA, PhD, MD)	8 (67%)
Some college/Technical degree/AA	2 (17%)
College degree (BA/BS)	2 (17%)
Employment Status, n (%)	
Employed full time	8 (67%)
Retired	2 (17%)
On disability	2 (17%)
Marital Status, n (%)	
Married	9 (75%)
Never married	1 (8%)
In a committed relationship	1 (8%)
Divorced	1 (8%)
U.S. Geographical Region, n (%)	
Midwest	8 (67%)
South	2 (17%)
West	1 (8%)
Northeast	1 (8%)
Health Insurance Type, n (%)	
Employer-sponsored private insurance only	7 (58%)
Employer-sponsored private insurance and Medicare	2 (17%)
Medicare only	1 (8%)
Medicaid	1 (8%)
Medicare primary, other private insurance secondary	1 (8%)

**Table 2:** Participant transplant and clinical characteristics (*N* = 12)

Transplant or Clinical Characteristic	*n* (%)
Donor Type (most recent transplant), n (%)	
Living donor	6 (50%)
Deceased donor	5 (42%)
Missing	1 (8%)
Total Transplants, n (%)	
1 transplant	8 (67%)
2 transplants	3 (25%)
3 transplants	1 (8%)
Time since 1st transplant, n (%)	
< 1 year	1 (8%)
1–3 years	3 (25%)
3–5 years	1 (8%)
5–10 years	4 (33%)
> 10 years	3 (25%)
Time since 2st transplant, n (%)	
< 1 year	1 (8%)
1–3 years	1 (8%)
3–5 years	1 (8%)
5–10 years	1 (8%)
Time since 3st transplant, n (%)	
< 1 year	1 (8%)
Treatment adjustments or additions due to BKV, n (%)	
Decrease in calcineurin inhibitor (e.g., tacrolimus - Prograf, Envarsus XR, cyclosporine - Neoral)	7 (58%)
Decrease in antimetabolite (e.g., mycophenolate mofetil - CellCept, mycophenolic acid - Myfortic, azathioprine - Imuran)	7 (58%)
Intravenous immune globulin (IVIG)	2 (17%)
Cidofovir (Vistide)	2 (17%)
Quinolone antibiotics (e.g., levofloxacin - Levaquin, ciprofloxacin - Cipro)	1 (8%)
Experimental therapy (e.g., virus-specific T cells, BKV-specific antibodies)	1 (8%)
Other (anti-viral medication)	1 (8%)
Outcomes as a result of BK-virus, n (%)	
Ongoing BKV	7 (58%)
Resolution of BKV	5 (42%)
Rejection	2 (17%)
Worsening kidney function	1 (8%)
Kidney failure	1 (8%)
Comorbidities, n (%)	
None	4 (33%)
Lung disease (e.g., COPD, asthma)	3 (25%)
Other mental health conditions	3 (25%)
Diabetes	2 (17%)
High blood pressure (hypertension)	2 (17%)
Other physical health conditions	2 (17%)
Autoimmune disease (e.g., lupus, rheumatoid arthritis)	2 (17%)
Other	2 (17%)
Heart disease	1 (8%)
Depression or anxiety	1 (8%)
Osteoporosis	1 (8%)
Self-reported ECOG Status, n (%)	
0 = I have normal activity, and no symptoms	8 (67%)
1 = I have some symptoms, but they do not require that I rest during the day	2 (17%)
2 = My condition requires me to rest for less than 50% of the day	2 (17%)
3 = My condition requires me to rest for more than 50% of the day	0 (%)
4 = I am unable to get out of bed	0 (%)

**Table 3:** Frequency, ratings, and example quotes for salient impacts related to disease education

Theme / Subtheme and Definition	*n* (%) reporting	*n* (%) rating; mean (range)	*n* (%) rating as highest*	Example quote
Disease education (Discussion of presence, quality, absence of patient education or provider education regarding the BK virus)	12 (100%)	4 (33%); 4.8 (3–6)	0 (0%)	“I wish they talked about it more, pre-transplant. And I know they can’t talk about every single thing, but this is major. BK and CMV are major things that I think they should talk about before you’re transplanted, to let you know, ‘This could happen.’ And not only, ‘This could happen,’ but ‘This is what it is, and this is what it does. And these are the symptoms, and this is how we’ll treat it.’” **(Participant 6)**
Need to educate self (Need to conduct self-guided research on BKV due to no or deficient disease education at the time of diagnosis)	6 (50%)	3 (25%); 5.3 (5–6)	0 (0%)	“Literally, it caused me to lose sleep because I spent hours and hours and hours researching this stuff on the internet. I mean, I saved and printed…let’s say 30 or 40 journal articles over the last 10 years […] I did not learn any of this from any doctor anywhere.” **(Participant 2)**

*During the ratings discussion, participants may give their highest ratings to one or more impacts

**Table 4:** Frequency, ratings, and example quotes for salient impacts related to emotional impacts

Theme / Subtheme and Definition	*n* (%) reporting	*n* (%) rating; mean (range)	*n* (%) rating as highest*	Example quote
Emotional Impacts (Discussion of any emotional effects [positive, negative, or absence of] related to any aspect of the participant’s experience with BKV)	11 (92%)	11 (92%); 6.3 (1–10)	8 (67%)	“[Being diagnosed with BKV is] like getting a birthday cake that you can’t eat. You’re excited about the birthday, and they bring the cake out, but you can’t eat it just because some reason. And, you’re excited. You wanna get to that birthday cake. But, hold on. Not yet. So, I couldn’t enjoy my kidney like I wanted to because of something that came up.” **(Participant 3)**
Fear of Graft Rejection (Fear or worry regarding potential rejection of the transplanted kidney)	11 (92%)	6 (50%); 7.7 (6–10)	3 (25%)	“Emotionally?…Almost traumatic stress, like if I think about it, I would never want that to come back in my blood again because it’s like you are in the situation that you cannot – there’s no treatment available… it was very stressful, and then you see your future as there is…hopelessness, I would say. If it happens this way, what is going to happen in the future? Like there’s no plan for the future. If it eventually affects the graft, then…” **(Participant 9)**
Stress or frustration (Relating to the need or complexity for additional monitoring or treatment beyond what was expected)	8 (67%)	3 (25%); 5.5 (2–10)	1 (8%)	“I think that it just sort of played into this whole feeling of being vulnerable post-transplant and lacking control over a lot of things. Having to worry about things that other people don’t have to worry about or think about. The whole idea is that you have the virus living inside you, and it’s usually harmless. However, now that you’re a transplant recipient, you’re in danger from it. It’s kind of a microcosm of the whole transplant experience and being more vulnerable afterward…It’s part of the overall stress of being a transplant recipient.” **(Participant 8)**
Uncertainty about treatment (Uncertainty or concern about duration, efficacy, side effects, or other aspects of BKV treatment)	8 (67%)	3 (25%); 5.3 (2–7)	2 (17%)	“**Interviewer**: Then medication changes, you went up to a 7. So, why did you rate that one the highest? **Participant 008**:I think because it felt like it was introducing more uncertainty about the health of the transplant itself and possibly having complications from these complications. Yeah, it felt very disappointing that the only way to address this was really to make these adjustments that could have other negative impacts.”
Emotional impact on friends and family (Emotional impact on social supports such as family and friends, such as fear or worry)	7 (58%)	4 (33%); 5.4 (1–10)	1 (8%)	“I think not just me, but the impact that it had on my family. My wife. My mom. My dad. […] There were so many unknowns that we had at that time, it just caused so much fear because we did not know what BK was beforehand.” **(Participant 3)**
Anxiety about lab tests (Anxiety, fear, or worry experienced prior, during or after lab tests for BK virus levels in blood)	6 (50%)	3 (25%); 7.3 (5–8)	1 (8%)	“I usually do my labs on Thursday, and I get the results Friday morning. So, when I see that notification – I get a panic attack: ‘Oh my God, what’s gonna show up in the lab? Has it gone down? Has it gone up?’ That stress and that panic thing I get… that feeling that I get when I see that notification. Yeah, so that was really bad.” **(Participant 9)**

*During the ratings discussion, participants may give their highest ratings to one or more impacts

**Table 5:** Frequency, ratings, and example quotes for salient impacts related to life disruption

Theme / Subtheme and Definition	*n* (%) reporting	*n* (%) rating; mean (range)	*n* (%) rating as highest*	Example quote
Life Disruption (Discussion of any disruptions of personal finances, work/school, time spent, or travel related to any aspect of the participant’s experience with BKV)	11 (92%)	10 (83%) 6.3 (1–10)	5 (42%)	“The thing is BK virus is very lab specific when you test them. And that’s why I only do testing at two labs, [in different states] so that they have continuity to look at when they make treatment decisions. So, that, I can go back and say the amount of time I spent going to the hospitals and getting infusions, the flying, because I fly into [US Southwest Hospital], […] so spending the time, what could I have done with all the time, money? So, I look back and say, ‘Oh my god, what is that energy that has been wasted on this?’ There’s so much.”
Time (BKV treatment, monitoring, or direct outcomes affecting how participants spend their time, including time lost, wasted, or misspent)	10 (83%)	10 (83%); 6.3 (1–10)	3 (25%)	“I’ve gotta call this doctor, I’ve gotta go get these labs, I need to look at my phone and see if my labs are back, or I wonder if the nurse emailed me yet… It’s always something in your mind that you need to do to manage your disease… people expect you to be doing all the normal things: working, being able to keep up with your job, not falling behind, keeping your house clean, doing all the things that you have to do, but you still have this stuff in the background and it takes up a lotta time.” **(Participant 10)**
Work (BKV treatment, monitoring, or direct outcomes affecting work, school, or regularly-scheduled work-like activity)	9 (75%)	3 (25%); 4.7 (1–7)	0 (0%)	“At that time, I was a teacher. And, of course, I had to take days off of work… get a sub and all those things… my health kept me away from my job, and that bothered me mentally because I loved what I did, and being around the young people meant a lot to me.” **(Participant 3)**
Financial (BKV treatment, monitoring or direct outcomes affecting participants’ budget or overall finances)	5 (42%)	4 (33%); 5.2 (4–9)	0 (0%)	“There was a big battle between [my insurer], [my doctor], [the pharmacy]. It was supposed to be approved, but they weren’t sharing information with each other properly so there was this huge thing where I had $200,000.00 of bills for six months that was not resolved. How much of this am I gonna have to pay?” **(Participant 2)**
Travel (BKV treatment, monitoring or direct outcomes affecting participants’ ability to travel for leisure, or requiring extra travel for care)	5 (42%)	3 (25%); 9 (7–10)	2 (17%)	“And obviously, I couldn’t take long trips because I have to do the labs every two weeks. And I have to be near one of these two centers. So, that’s a limitation.” **(Participant 1)**

*During the ratings discussion, participants may give their highest ratings to one or more impacts

**Table 6:** Frequency, ratings, and example quotes for salient impacts related to the experience of BKV care

Theme / Subtheme and Definition	*n* (%) reporting	*n* (%) rating; mean (range)	*n* (%) rating as highest*	Example quote
Experience of BKV Care (Any discussion of navigation of health care, treatment options and insurance coverage of treatments or monitoring. This also includes navigating conflicting lab values and/or advice from clinicians and discussion of needing to independently find care elsewhere (requiring self-advocacy)	8 (67%)	4 (33%) 5.6 (2–10)	1 (16%)	“[when scheduling my routine labs labs, I’ll ask] “All right, I’m coming in on Wednesday to get labs drawn. Do you want a BK with that?” …And then that won’t get done. And then I’ll be back in the hospital. “Do you want that BK?” And then it won’t get done. And then finally I’ll go back to home care. They’ll finally have it set up with home care after three weeks…I’m typically very frustrated with the kidney coordination… they’re just not very skilled at keeping track of the details.” **(Participant 11)**

*During the ratings discussion, participants may give their highest ratings to one or more impacts

**Table 7:** Frequency, ratings, and example quotes for salient impacts related to patient-clinician communication

Theme / Subtheme and Definition	*n* (%) reporting	*n* (%) rating; mean (range)	*n* (%) rating as highest*	Example quote
Patient-clinician communication (Any discussion of navigation impactful communication between the participant as a patient and member[s] of their treatment team)	9 (75%)	4 (33%) 4 (2–5)	1 (16%)	“What made me upset was not knowing that that’s a possibility, okay? What really baffled me was the fact that that one doctor that came in, and he says, ‘Well, I think they just over-medicated you. They over suppressed your system because if they wouldn’t have done that, you probably wouldn’t have got the BK virus.’ I don’t know how true that could be… so who actually had the BK virus? What kidney had the BK virus? Was it my kidney? Or was it the transplant kidney? And I asked, and they said they don’t know.” **(Participant 12)** “And that’s when [the time of diagnosis] they basically just downplayed. They said, ‘Don’t be concerned. It’s okay.’ Even when it happened early, as I remember speaking with some of them including head of transplant, nephrology, there’s, ‘Yeah, [participant name] it may sound concerning to you, but don’t worry. We’ve done this so many times.’ I mean, I think they were trying to put me at ease in a way, but inside, that wasn’t quite comforting.” **(Participant 1)**

*During the ratings discussion, participants may give their highest ratings to one or more impacts

**Table 8:** Frequency, ratings, and example quotes for salient impacts related to physical impacts

Theme / Subtheme and Definition	*n* (%) reporting	*n* (%) rating; mean (range)	*n* (%) rating as highest*	Example quote
Physical Impacts (Discussion of any physical symptoms or side effects related to BKV monitoring or treatment)	7 (58%)	6 (50%) 7.3 (1–10)	3 (25%)	“For me, the symptoms of BKV are like having problems on your roof. Because you can’t really see your roof, and there’s no intermediate thing you can do about your roof, and then your roof starts to leak… I’m at that stage where my roof is damaged, but it hasn’t started to leak yet.” **(Participant 11)**
Treatment-related (Discussion of any physical symptoms or clinical outcomes that the participant attributes to treatment for their BKV)	7 (58%)	4 (33%); 7.5 (1–10)	3 (25%)	**Interviewer**:So, you said that the physical impacts, or the side effects from the medications, you ranked that as the highest. You said an 8 or a 9… Why did you rank that one the highest for you? **Participant 1**: It’s weakness. So, there are certain days I feel weak…muscular weakness, muscular pains.
Disease-related (Discussion of clinician-diagnosed or diagnosable conditions or physical symptoms directly caused by BKV or any physical symptoms that the participant attributes to the BK virus)	6 (50%)	5 (42%); 7.6 (5–10)	1 (8%)	“For some odd reason, when the copies were increased, I experienced really bad diarrhea… I wasn’t able to get out as much. Even when it came to traveling for advocacy… because of the diarrhea, it was bad.” **(Participant 5)**

## Supplementary Information

Below is the link to the electronic supplementary material.

Supplementary Material 1

## Data Availability

The datasets generated and analyzed during the current study are not publicly available due to risk of participant re-identification. Although all data were de-identified, the qualitative nature of the transcripts and analysis files may still contain potentially identifiable information. As such, sharing these materials would compromise patient confidentiality. Researchers with specific questions about the study may contact the corresponding author for more information.

## Abbreviations

AMR: Antibody-mediated rejection
BKV: BK Virus
BKVN: BK virus-associated nephropathy
COREQ: Consolidated Criteria for Reporting Qualitative Research
HRQoL: Health-Related Quality of Life
IS: Immunosuppressive(s)
PRO: Patient-reported outcome
US FDA: United States Food and Drug Administration
