# Supplementary material for: Development of a conceptual model of BKV impacts on health-related quality of life in kidney transplant recipients: a qualitative study
Source: J Patient Rep Outcomes. 2026 Jan 7;10:4. doi: 10.1186/s41687-025-00987-x (PMC12789305; doi:10.1186/s41687-025-00987-x)
Supplement: Supplementary file 1 — Supplementary Material 1 [file 41687_2025_987_MOESM1_ESM.docx]

# Supplemental Files

## **Interview Guide Development**

This section details the key refinements made to the interview guide based on feedback from Memo Patient Advocacy Board (PAB) research partners and insights gained from preliminary interviews.

### **Research Partners’ Input on the Interview Guide**

In June and July 2024, PAB representatives with lived BKV experience after kidney transplant reviewed the draft interview guide developed by the NU team and provided insights and suggestions. Key areas of feedback and corresponding refinements are detailed below:

- PAB representatives emphasized regional variations in BKV management and patient experiences.
- PAB representatives pointed out that pre-transplant education about BKV is often insufficient, especially for recipients of deceased donor kidneys.
- PAB representatives noted that both patients and providers might misattribute BKV symptoms to medication side effects, leading to delayed diagnosis.
- To improve the logical flow of the interview, a research partner suggested reordering some interview probes.

The NU study team then carefully considered and incorporated this feedback into the interview guide, further refining the questions and structure to better capture the impacts of BKV after a kidney transplant.

### **Preliminary Interview Guide Testing and Refinement**

The NU team conducted three preliminary interviews (August and September 2024), informing several refinements to the interview guide. One qualitative researcher (CH) with extensive experience in qualitative methodologies and interviewing, conducted the preliminary interviews, while other three researchers (SS, MK, EJ) listened. The guide and interview methods were further refined based on these interviews:

**REDCap form:** To streamline data collection, the NU team implemented a REDCap form to gather baseline information, including clinical history, transplant details, and demographic information. This aimed to reduce the length of the interviews, which were one hour and 30 minutes long, and, therefore, avoid potential participant (and interviewer) fatigue.

**Interview Guide Content Refinement:** The team revised prompts and added targeted probes to improve capture of patient-reported psychosocial impact. These addressed broader social and relational dimensions, emotional impacts, caregiver experiences, and daily life disruptions for both patients and care partners. Furthermore, noticing that participants often shared experiences about logistical challenges in navigating the healthcare system, the team added probes to address issues such as managing insurance and coordinating treatments for all participants.

**Interview Guide Structure Refinement:** The interview guide was reordered to follow a more logical sequence of BKV diagnosis and treatment, starting with an open-ended question (“*Tell me about how you were diagnosed with BKV.*”) and moving to more specific questions based on responses from the participant. Questions about pre-transplant education were moved earlier, while questions about emotional and daily impacts were positioned later, after covering diagnosis and treatment basics.

**Analogy Inclusion:** Participants were asked to identify an analogy to describe their experience with BKV diagnosis, symptoms, and treatment in order to help participants express their BKV experience by connecting them to more familiar experiences and stimulating and encouraging them to engage more thoughtfully.

### **Finalization of Interview Guide**

The final interview guide covered the following main topics:

1. **Diagnosis experience:**

- Pre-transplant knowledge of BKV risk
- Story of BKV diagnosis (how, by whom, when)
- Thoughts and feelings when diagnosed with BK Viremia or BKVAN
- Family or support system reactions to the diagnosis

1. **Symptoms and Impacts:**

- Physical symptoms related to BKV
- Impacts on quality of life, including emotional, financial, and social aspects

1. **Monitoring and Treatment:**

- Next steps in care after BKV diagnosis
- Experience with monitoring and treating BKV

1. **Outcomes/resolution:**

- Outcomes after BKV treatment
- Most important impacts to the patient

1. **General**

- Advice for future kidney transplant recipients about BKV
- Advice for those recently diagnosed with BKV
- Additional experiences or information about BKV that we did not mention

Throughout the guide, interviewers are instructed to probe for details on timing, frequency, severity, duration, and impacts on emotional, financial, social, and caregiver aspects of the participant's life.

# Supplement 2

**Consolidated criteria for reporting qualitative studies (COREQ): 32-item checklist**

| **Domain 1: Research team and reflexivity** | | |
| --- | --- | --- |
| *Personal characteristics* | | |
| 1. | Interviewers | CH and EJ completed all study interviews. |
| 2. | Credentials | CH has an MSW; EJ has an MPH. |
| 3. | Occupation | JDP, MK, and SS are faculty-level HRQoL investigators in a medical school. CH and EJ are project staff and qualitative research analysts. |
| 4. | Gender | Both interviewers are female. |
| 5. | Experience and training | SS and CH are experienced qualitative researchers. EJ was trained to conduct interviews on this project and was closely supervised, including shadowing, reverse shadowing, and regular debriefing following interviews with CH. |
| *Relationship with participants* | | |
| 6. | Relationship established | The interviewers had no prior relationships with study participants but established rapport during the recruitment and consent process. |
| 7. | Participant knowledge of interviewer | Participants did not know the interviewer prior to the day of recruitment. |
| 8. | Interviewer characteristics | (Described in Items 2, 3, 4, and 5) |
| **Domain 2** | | |
| *Theoretical framework* | | |
| 9. | Methodological orientation and theory | Thematic analysis with constant comparison |
| *Participant selection* | | |
| 10. | Sampling | Purposive sampling method |
| 11. | Method of approach | A transplant nephrologist reviewed medical records at a large academic cancer center in the Midwest US to identify patients with a confirmed diagnosis of BK viremia or BK nephropathy. A research partner and patient advocate identified potential participants from their advocacy network and obtained their permission before forwarding contact information to the study team. Members of the Memo Therapeutics Patient Advisory Board (PAB) were also invited to participate.  Individuals were contacted by telephone or email by a study team member, who explained the study using an IRB-approved recruitment script and obtained written informed consent from interested patients. |
| 12. | Sample size | A total of 12 participants were in this study |
| 13. | Non-participation | 25 individuals were approached to enroll in the study. Eleven individuals were nonresponsive or lost to follow-up. Three individuals did not speak English and were therefore, ineligible to participate. No participants declined enrollment or dropped out of the study after consenting. |
| *Setting* | | |
| 14. | Setting of data collection | Virtual interviews with video and audio were held on Zoom teleconferencing platform. |
| 15. | Presence of non-participants | One participant had a spouse present in the background for a portion of the interview, though the spouse did not contribute to any of the main discussion between participant and interviewer. |
| 16. | Description of sample | Mean age of the 12 patients who completed concept elicitation interviews was 48 years. The most commonly reported race or ethnicity was Black or African American (n=4), followed by White or Asian (n=3, respectively). Seven participants were female. Most participants (n=8) had a college-level or advanced degree. Most participants (n=8) were employed full-time. |
| *Data collection* | | |
| 17. | Interview guide | The guide was modeled after prior research with similar aims and iteratively refined based on patient feedback and early interviews. |
| 18. | Repeat interviews | There were no repeat interviews. |
| 19. | Audio/visual recording | Interviews were audio-recorded, transcribed verbatim, and de-identified prior to analysis. |
| 20. | Field notes | Interviewers took detailed field notes during the interview. After the interview, field notes entered into an Excel file for each study participant. |
| 21. | Duration | The interviews lasted approximately 90 minutes each. |
| 22. | Data saturation | Saturation was assessed by evaluating the point at which no new relevant themes emerged for three consecutive interviews. Saturation occurred after the fifth interview. An additional seven interviews were completed to ensure comprehensive capture from a diverse sample. |
| 23. | Transcripts returned | Transcripts were not returned to participants. |
| **Domain 3: Analysis and findings** | | |
| *Data analysis* | | |
| 24. | Number of data coders | Two data analysts independently reviewed and coded three transcripts using the comment function in Microsoft Word and the using the draft codebook, noting missing or problematic codes. In group meetings, the coded transcripts were collectively reviewed, discrepancies were discussed, and the codebook was refined. After finalizing the codebook, two team members independently coded the remaining transcripts. |
| 25. | Description of the coding tree | First, detailed field notes were reviewed and lists of quality-of-life impacts were compiled and redundant concepts were removed, resulting in a preliminary codebook. The codebook was refined iteratively during analysis via team discussion. |
| 26. | Derivation of themes | Themes were derived inductively from the data. |
| 27. | Software | Dedoose |
| 28. | Participant checking | Participants did not provide feedback on the research results. |
| *Reporting* | | |
| 29. | Quotations presented | Quotations are included in the manuscript. |
| 30. | Data and findings consistent | There is consistency between the data and the research findings. |
| 31. | Clarity of major themes | Major and minor themes are clearly delineated in the Results and Discussion section of the manuscript. |

S1. Support System Characteristics (N=12)

| Participant Support System Characteristic | n (%) |
| --- | --- |
| Having a care partner(s), caregiver(s), n (%) |  |
| Yes | 9 (75%) |
| No | 3 (25%) |
| Relationship with care partner, n (%) |  |
| Spouse/Partner | 9 (75%) |
| Multiple family members | 6 (17%) |
| Children involved in care | 2 (17%) |
| Friends/broader social network | 1 (8%) |
| Types of support received, n (%) |  |
| Emotional support (e.g., companionship, encouragement, comfort) | 8 (67%) |
| Transportation assistance (e.g., driving to doctors appointments, providing transportation to attend non-medical activities) | 5 (42%) |
| Assistance with medication management (e.g. picking up medication from a pharmacy, organizing medication, overseeing/tracking medication | 3 (25%) |
| Help with daily activities (e.g., cooking, cleaning)) | 3 (25%) |
| Information gathering about BKV (e.g., researching medical information) | 2 (17%) |
| Financial support (e.g., paying bills, providing financial assistance)) | 2 (17%) |
| Coordination of medical care (e.g., scheduling medical appointments, communicating and following up with healthcare providers, helping with insurance claims)) | 1 (8%) |

S2. Overall frequency and frequency, mean, and range of HRQoL ratings of Disease Education subthemes

| Subtheme | Definition | n (%)  reporting^1^ | n (%)  rating; mean (range) | n (%) rating as most impactful^2^ |
| --- | --- | --- | --- | --- |
| No pre-transplant education on BKV | A reported lack of knowledge or education about the risk of BKV prior to transplant | 10 (83%) | 2 (16%);  4.0 (3-5) | 0 (0%) |
| Need to educate self | Need to conduct self-guided research on BKV due to no or deficient disease education at the time of diagnosis | 6 (50%) | 3 (25%);  5.3 (5-6) | 0 (0%) |
| No educational materials provided | Participant indicated they were not given any written educational materials at the time of diagnosis | 5 (42%) | 0 (0%) | 0 (0%) |

S3. Overall frequency and frequency, mean, and range of HRQoL ratings of Emotional Impacts subthemes

| Subtheme | Definition | n (%)  reporting^1^ | n (%)  rating; mean (range) | n (%) rating as most impactful^2^ |
| --- | --- | --- | --- | --- |
| Fear of Graft Rejection | Fear or worry regarding potential rejection of the transplanted kidney | 11 (92%) | 6 (50%);  7.7 (6-10) | 3 (25%) |
| Stress or frustration | Expressed stress or frustration relating to the need or complexity for additional monitoring treatment, or outcomes beyond what was expected | 8 (67%) | 3 (25%);  5.5 (2-10) | 1 (8%) |
| Uncertainty about treatment | Uncertainty or concern about duration, efficacy, side effects, or other aspects of BKV treatment | 8 (67%) | 3 (25%);  5.3 (2-7) | 2 (17%) |
| No control or no cure | Distress over no cure for BKV or limited options to control virus spread; Distress over a loss of perceived control | 8 (67%) | 2 (17%);  5.0 (2-8) | 1 (8%) |
| Emotional impact on friends and family | Emotional impact on social support network, such as fear or worry | 7 (58%) | 4 (33%);  5.4 (1-10) | 1 (8%) |
| Anxiety about lab tests | Anxiety, fear, or worry experienced prior, during or after lab tests for BK virus levels in blood | 6 (50%) | 3 (25%);  7.3 (5-8) | 1 (8%) |
| Scary | Reports of feeling scared or generally fearful, not specific to graft loss, shortened lifespan, or reinfection | 6 (50%) | 1 (8%);  10 (no range) | 1 (8%) |
| Overwhelmed, helpless, drained | Emotional responses related to BKV described as overwhelming, draining, or a sense of helplessness | 6 (50%) | 1 (8%);  8 (no range) | 0 (0%) |
| Dread | Apprehensive anticipation regarding any aspect of the BKV experience | 6 (50%) | 0 (0%) | 0 (0%) |
| Not meeting post-transplant health expectations | Discussion of how BKV has delayed or is preventing realization of a healthier life after kidney transplant | 5 (42%) | 2 (17%);  6.5 (6-7) | 0 (0%) |
| Reduced or lack of confidence in treatments and doctors | Discussion of reduced confidence in the state of the current standard of care of BKV or clinicians’ ability to treat BKV | 5 (42%) | 2 (17%);  6 (4-8) | 2 (17%) |
| Fear about stopping immunosuppression | Anxiety or fear over reducing or stopping anti-rejection medications to treat BK virus | 4 (33%) | 2 (17%);  7 (no range) | 1 (8%) |
| Depressing | Feelings of depression, sadness, hopelessness | 4 (33%) | 1 (8%);  8 (no range) | 0 (0%) |
| Wasted effort | Feeling like the effort to obtain, or the transplant was wasted | 4 (33%) | 1 (8%);  6 (no range) | 0 (0%) |
| Shock, surprise, or stun | BKV diagnosis or treatment was unexpected, causing feelings of shock or negative surprise | 4 (33%) | 1 (8%);  5.5 (5-6)^a^ | 1 (8%) |
| Dynamic with donor | BKV diagnosis affected interpersonal dynamics with known donor, e.g., concern, fear, resentment | 3 (25%) | 2 (17%);  5.3 (3-7) | 0 (0%) |
| Impatience | General feelings of impatience around BKV diagnosis, treatment, and clinical improvement | 3 (25%) | 1 (8%);  6 (no range) | 0 (0%) |
| Added to stress of post-transplant | BKV infection complicated and added to post-transplant stress | 3 (25%) | 1 (8%);  3 (no range) | 0 (0%) |
| Fear of recurrence | Fear of BKV infection occurring within the same transplant, or in subsequent transplants | 3 (25%) | 0 (0%) | 0 (0%) |
| Inability to live a normal life | Expressed concern that BKV will cause participant to become unable to live a normal life post-transplant | 3 (25%) | 0 (0%) | 0 (0%) |
| Fear of shortened lifespan | Fear that BKV will end life prematurely | 2 (18%) | 2 (17%);  9 (8-10) | 2 (17%) |
| Feeling different to others with BKV | Due to a complicated course of BKV, feeling unable to relate to others in transplant community who have had BKV | 2 (18%) | 0 (0%) | 0 (0%) |
| Feeling different to others with kidney transplants | Feeling unable to relate to others in transplant community who **have not** been diagnosed with BKV | 2 (18%) | 0 (0%) | 0 (0%) |
| Self-pity | Self-described excessive unhappiness over BKV or asking self, “Why me?” | 2 (18%) | 0 (0%) | 0 (0%) |
| Slow process to understand improvement | Emotional burden of waiting for clinical improvement | 2 (18%) | 0 (0%) | 0 (0%) |

^1^One participant each identified the following Emotional Impact subthemes: Persistent doubt; Emotional isolation from family/friends

^2^During the ratings discussion, participants may give their highest ratings to one or more impacts

S4. Overall frequency and frequency, mean, and range of HRQoL ratings of Life Disruption subthemes

| Subtheme | Definition | n (%)  reporting | n (%)  rating; mean (range) | n (%) rating as most impactful^1^ |
| --- | --- | --- | --- | --- |
| Time | BKV treatment, monitoring, or direct outcomes affecting how participants spend their time, including time lost, wasted, or misspent | 10 (83%) | 10 (83%);  6.3 (1-10) | 3 (25%) |
| Work | BKV treatment, monitoring, or direct outcomes affecting work, school, or regularly-scheduled work-like activity | 9 (75%) | 3 (25%); 4.7 (1-7) | 0 (0%) |
| Financial | BKV treatment, monitoring or direct outcomes affecting participants’ budget or overall finances | 5 (42%) | 4 (33%);  5.2 (4-9) | 0 (0%) |
| Travel | BKV treatment, monitoring or direct outcomes affecting participants’ ability to travel for leisure, or requiring extra travel for care | 5 (42%) | 3 (25%);  9 (7-10) | 2 (17%) |

^1^During the ratings discussion, participants may give their highest ratings to one or more impacts

S5. Overall frequency and frequency, mean, and range of HRQoL ratings of Experience of BKV Care subthemes

| Subtheme | Definition | n (%)  reporting | n (%)  rating; mean (range) | n (%) rating as most impactful |
| --- | --- | --- | --- | --- |
| Care inconsistency | Discussions of conflicting lab values, treatment approaches, or advice from clinicians | 7 (58%) | 2 (17%);  4.5 (2-7) | 0 (0%) |
| Insurance | Discussions of insurance coverage for care including navigation of this coverage | 3 (25%) | 2 (17%);  7 (4-10) | 1 (8%) |
| Elsewhere Care | Discussions of the need to independently find care outside the participant’s transplant center, requiring a degree of self-advocacy and advanced care coordination by the patient | 2 (17%) | 1 (8%);  5 (no range) | 0 (0%) |

S6. Overall frequency and frequency, mean, and range of HRQoL ratings of Physical Impacts subthemes

| Subtheme | Definition | n (%)  reporting^1^ | n (%)  rating; mean (range) | n (%) rating as most impactful^2^ |
| --- | --- | --- | --- | --- |
| Treatment-related | Discussion of any physical symptoms or clinical outcomes that the participant attributes to treatment for their BKV | 7 (58%) | 4 (33%);  7.5 (1-10) | 3 (25%) |
| Disease-related | Discussion of clinician-diagnosed or diagnosable conditions or physical symptoms directly caused by BKV or any physical symptoms that the participant attributes to the BK virus | 6 (50%) | 5 (42%);  7.6 (5-10) | 1 (8%) |
| Graft rejection caused by immunosuppression | Discussions of any degree of graft rejection described by participant as caused by their immune suppression reduction to treat BKV | 3 (25%) | 1 (8%);  8 (no range) | 1 (8%) |
| Fatigue | Discussions of fatigue or tiredness that participant attributes to BK virus infection | 3 (25%) | 1 (8%);  7 (no range) | 0 (0%) |
| Discomfort with needles during antiviral treatment | Discussion of pain or discomfort with intravenous antiviral treatments | 3 (25%) | 0 (0%) | 0 (0%) |
| Muscle loss or weakness | Discussion of muscle loss or muscle weakness that participant attributes to treatment for BKV | 2 (17%) | 2 (17%);  9 (no range) | 1 (8%) |
| Steroids and hyperglycemia | Discussion of steroids causing hyperglycemia or diabetes | 2 (17%) | 2 (17%);  7 (8-9) | 1 (8%) |
| Flu-like symptoms | Body pain, fever, fatigue that participant attributes to BKV infection | 2 (17%) | 0 (0%) | 0 (0%) |
| Headaches | Discussion of headaches caused by antiviral treatment for BKV | 2 (17%) | 0 (0%) | 0 (0%) |

^1^One participant each identified the following Physical Impact subthemes: Weight loss; Diarrhea; Discomfort in kidney; Hypertension

^2^During the ratings discussion, participants may give their highest ratings to one or more impacts

S7. Overall frequency and frequency, mean, and range of HRQoL ratings of Patient-Clinician Communication subthemes

| Subtheme | Definition | n (%)  reporting^1^ | n (%)  rating; mean (range) | n (%) rating as most impactful |
| --- | --- | --- | --- | --- |
| Ineffective BKV-related communication from clinician | Communication with clinician was not reassuring or led to misunderstandings | 5 (42%) | 0 (0%) | 0 (0%) |

^1^One participant each identified the following subtheme: Received inconsistent guidance on immunosuppression
